# Dataset on the mass spectrometry-based proteomic profiling of the kidney from wild type and the dystrophic *mdx-4cv* mouse model of X-linked muscular dystrophy

**DOI:** 10.1016/j.dib.2019.105067

**Published:** 2020-01-02

**Authors:** Paul Dowling, Margit Zweyer, Maren Raucamp, Michael Henry, Paula Meleady, Dieter Swandulla, Kay Ohlendieck

**Affiliations:** a Department of Biology, Maynooth University, National University of Ireland, Maynooth, W23F2H6, Co. Kildare, Ireland; b Kathleen Lonsdale Institute for Human Health Research, Maynooth University, Maynooth, W23F2H6, Co. Kildare, Ireland; c Institute of Physiology II, University of Bonn, D53115, Bonn, Germany; d National Institute for Cellular Biotechnology, Dublin City University, Dublin 9, Ireland

**Keywords:** Kidney proteome, Mass spectrometry, Muscular dystrophy, Proteomics

## Abstract

The proteomic data presented in this article provide supporting information to the related research article “Proteomic and cell biological profiling of the renal phenotype of the *mdx-4cv* mouse model of Duchenne muscular dystrophy” (Dowling et al., 2019) [1]. This article supplies additional datasets on protein species with increased versus decreased concentration in the kidney from the dystrophic *mdx-4cv* mouse, as well as tables with mass spectrometrically identified kidney marker proteins that exhibit characteristic tissue distributions, subcellular localizations and physiological functions. Information is provided on the underlying multi-consensus protein listings from the proteomic screening of both wild type and *mdx-4cv* mouse kidneys. The data article provides comprehensive information on the systematic and mass spectrometric identification of the mouse kidney proteome.

**Table:** Specifications Table

Subject	Biochemistry
Specific subject area	Biomedicine
Type of data	Tables
How data were acquired	LC-MS/MS analysis, using an Orbitrap Fusion Tribrid Mass Spectrometer (Thermo Scientific)
Data format	Raw data and analysed data
Parameters for data collection	Renal protein was extracted from whole kidneys from wild type and dystrophic *mdx-4cv* mice.
Description of data collection	Comparative mass spectrometry-based proteomic profiling of kidney extracts
Data source location	Maynooth, Co. Kildare, Ireland
Data accessibility	The data on kidney marker proteins are available with this article. The multi-consensus file and raw data files of all identified kidney proteins is available through a public repository. Repository name: Open Science Framework Data identification number: bz3kv Direct URL to data: https://osf.io/bz3kv/
Related research article	Paul Dowling, Margit Zweyer, Maren Raucamp, Michael Henry, Paula Meleady, Dieter Swandulla and Kay Ohlendieck Proteomic and cell biological profiling of the renal phenotype of the mdx-4cv mouse model of Duchenne muscular dystrophy European Journal of Cell Biology (2019) (available online November 18, 2019, 151059) https://doi.org/10.1016/j.ejcb.2019.151059

**Table:** 

**Value of the Data** • Mass spectrometric data presented in this article provide a detailed listing of the protein isoforms of the assessable mouse kidney proteome. • Proteomic data provide a summary of changed renal proteins in the dystrophic *mdx-4cv* mouse model of X-linked muscular dystrophy. • The proteomic data give an overview of the identification of tissue-specific kidney marker proteins. • The mass spectrometric data are valuable to serve as a comprehensive repository of the mouse proteome for comparative biochemical studies.

## 1 Data

This data paper presents the findings from the mass spectrometric profiling of mouse kidney extracts, and relates to the comparative survey of the renal proteome from wild type versus the dystrophic *mdx-4cv* mouse model of Duchenne muscular dystrophy [1]. With the help of an Orbitrap Fusion Tribrid mass spectrometer, 5878 protein species were identified in renal tissue extracts. The multi-consensus file of mouse kidney that contains data from the analysis of 28 separate mass spectrometric sample runs, as well as the raw data underlying the cataloguing of the kidney, has been deposited to the Open Science Framework under the project title ‘Proteomic profiling of mouse kidney’; Link: https://osf.io/bz3kv/(Date created: 2018-10-04; Date made public: 2019-12-02). The analyses were carried out with 2 technical repeats of 7 biological repeats of 12-month old wild type kidney extracts, as well as 2 technical repeats of 7 biological repeats of 12-month old *mdx-4cv* kidney extracts. In relation to the report by Dowling et al. [1], additional datasets on proteins with an increased versus decreased concentration in the kidney from the dystrophic *mdx-4cv* mouse are presented in Table 1, Table 2. Table 1 lists the mass spectrometric identification of kidney proteins with a less than 1.5-fold increase in the *mdx-4cv* mouse. Kidney proteins with a less than 1.5-fold increased concentration in the *mdx-4cv* mouse are presented in Table 2. The most abundant high-molecular-mass kidney proteins are presented in Table 3. Information on typical tissue-specific kidney marker proteins [[2], [3], [4], [5], [6]] is provided in Table 4. In Table 5 are listed mass spectrometrically identified kidney marker proteins with characteristic physiological functions in ion homeostasis, including specific isoforms of the sodium/calcium exchanger, sodium/glucose cotransporter, the plasma membrane calcium-transporting ATPase, the sodium/potassium-transporting ATPase, the endoplasmic reticulum calcium ATPase, calcium-binding proteins, voltage-dependent calcium channels and chloride channels [2]. In relation to the comparative survey of fatty acid binding proteins in the kidney from wild type versus the dystrophic *mdx-4cv* mouse model of dystrophinopathy [1], Table 6 lists the fatty acid binding proteins that were identified by mass spectrometry. This includes fatty acid-binding protein isoforms FABP1, FABP3, FABP4, FABP5 and FABP7 [7]. Kidney dystrophin and its associated proteins were identified in renal extracts, including dystrophin (Dp140), dystroglycan, alpha-1-syntrophin, beta-2-syntrophin, epsilon-sarcoglycan, alpha-dystrobrevin and beta-dystrobrevin [8], as listed in Table 7.

**Table 1:** Proteomic identification of kidney proteins with an increased abundance below 1.5-fold in the *mdx-4cv* model of Duchenne muscular dystrophy.

Accession	Protein	Gene	Unique peptides	Confidence score	Anova (p)	Max fold change
O35657	Sialidase-1	Neu1	2	4.6649	0.00419	1.49
O54990	Prominin-1	Prom1	2	13.8091	0.01535	1.49
Q91WU2	Solute carrier family 22 member 7	Slc22a7	2	5.6395	0.04080	1.49
D3Z7P3	Glutaminase kidney isoform, mitochondrial	Gls	3	12.9045	0.00653	1.48
P48758	Carbonyl reductase [NADPH] 1	Cbr1	4	17.5670	0.00074	1.48
Q61391	Neprilysin	Mme	4	12.3698	0.00261	1.48
G5E829	Plasma membrane calcium-transporting ATPase 1	Atp2b1	2	7.9881	0.00011	1.47
P24472	Glutathione S-transferase A4	Gsta4	2	5.6183	0.00201	1.47
O88343	Electrogenic sodium bicarbonate cotransporter 1	Slc4a4	7	22.6034	1.04E-08	1.47
Q9R257	Heme-binding protein 1	Hebp1	2	8.7884	0.00369	1.46
P61922	4-aminobutyrate aminotransferase, mitochondrial	Abat	6	21.809	4.63E-05	1.46
O70370	Cathepsin S	Ctss	2	9.0446	0.00011	1.45
P70172	Ileal sodium/bile acid cotransporter	Slc10a2	2	5.4973	0.00038	1.45
P24452	Macrophage-capping protein	Capg	2	5.5246	0.02331	1.45
P97449	Aminopeptidase N	Anpep	5	27.1718	0.00188	1.45
Q8BMS1	Trifunctional enzyme subunit alpha, mitochondrial	Hadha	2	6.4455	0.00174	1.44
Q9QXE0	2-hydroxyacyl-CoA lyase 1	Hacl1	2	9.9044	0.00925	1.44
Q9D687	Sodium-dependent neutral amino acid transporter B(0)AT1	Slc6a19	3	10.4909	1.27E-06	1.43
Q9D964	Glycine amidinotransferase, mitochondrial	Gatm	2	7.0537	0.00819	1.42
Q9Z306	Solute carrier family 22 member 4	Slc22a4	2	6.6313	0.00929	1.42
Q49B93	Sodium-coupled monocarboxylate transporter 2	Slc5a12	2	6.7141	1.69E-05	1.41
Q9CQ65	S-methyl-5′-thioadenosine phosphorylase	Mtap	2	5.9308	0.00323	1.41
P23780	Beta-galactosidase	Glb1	2	6.2503	0.00437	1.4
P24668	Cation-dependent mannose-6-phosphate receptor	M6pr	2	6.5894	0.04069	1.4
P09470	Angiotensin-converting enzyme	Ace	12	42.5194	0.00642	1.4
Q8VCZ9	Hydroxyproline dehydrogenase	Prodh2	2	6.1044	0.00172	1.39
P45376	Aldose reductase	Akr1b1	5	13.6831	0.00165	1.39
O88986	2-amino-3-ketobutyrate coenzyme A ligase, mitochondrial	Gcat	3	10.1459	4.10E-05	1.38
Q9WV92	Band 4.1-like protein 3	Epb41l3	4	14.7070	0.00046	1.38
Q9DBT9	Dimethylglycine dehydrogenase, mitochondrial	Dmgdh	5	18.8616	0.00028	1.38
O88428	Bifunctional 3′-phosphoadenosine 5′-phosphosulfate synthase 2	Papss2	11	37.6264	0.00095	1.38
O09164	Extracellular superoxide dismutase [Cu–Zn]	Sod3	2	11.3667	1.53E-05	1.37
O70493	Sorting nexin-12	Snx12	4	10.9785	6.57E-06	1.36
Q9Z1G3	V-type proton ATPase subunit C 1	Atp6v1c1	4	14.1960	0.00010	1.36
Q9R092	17-beta-hydroxysteroid dehydrogenase type 6	Hsd17b6	2	7.8166	0.00030	1.35
Q9EQ20	Methylmalonate-semialdehyde dehydrogenase [acylating], mitochondrial	Aldh6a1	2	7.8288	0.02301	1.35
Q8K157	Aldose 1-epimerase	Galm	3	10.5439	0.00726	1.35
P16406	Glutamyl aminopeptidase	Enpep	10	35.0875	1.14E-06	1.35
Q9QWR8	Alpha-N-acetylgalactosaminidase	Naga	3	8.1744	0.00102	1.34
Q9DCJ9	N-acetylneuraminate lyase	Npl	4	13.4008	0.00150	1.34
Q8R3P0	Aspartoacylase	Aspa	2	4.8961	5.94E-05	1.33
P24527	Leukotriene A-4 hydrolase	Lta4h	2	7.3695	0.00689	1.33
P16675	Lysosomal protective protein	Ctsa	2	7.8468	0.01961	1.33
Q00519	Xanthine dehydrogenase/oxidase	Xdh	3	11.0764	0.00741	1.33
P10649	Glutathione S-transferase Mu 1	Gstm1	4	16.5436	6.74E-05	1.33
Q91X52	l-xylulose reductase	Dcxr	5	22.2456	5.55E-07	1.33
P50431	Serine hydroxymethyltransferase, cytosolic	Shmt1	6	19.5642	0.00172	1.33
Q9CXN7	Phenazine biosynthesis-like domain-containing protein 2	Pbld2	3	13.0305	5.80E-07	1.32
P15626	Glutathione S-transferase Mu 2	Gstm2	3	10.7236	0.00011	1.32
Q99LB7	Sarcosine dehydrogenase, mitochondrial	Sardh	4	14.0763	6.64E-05	1.32
Q99MZ6	Unconventional myosin-VIIb	Myo7b	4	11.6505	0.00061	1.32
Q91Y97	Fructose-bisphosphate aldolase B	Aldob	5	22.2473	0.02101	1.32
P26443	Glutamate dehydrogenase 1, mitochondrial	Glud1	6	19.3021	2.20E-07	1.32
P48774	Glutathione S-transferase Mu 5	Gstm5	3	8.9950	3.80E-05	1.31
O88844	Isocitrate dehydrogenase [NADP] cytoplasmic	Idh1	3	8.7557	0.00071	1.31
Q62433	Protein NDRG1	Ndrg1	5	23.1403	1.78E-05	1.31
Q91VA0	Acyl-coenzyme A synthetase ACSM1, mitochondrial	Acsm1	5	19.1532	0.00374	1.31
P10493	Nidogen-1	Nid1	2	8.7788	0.00333	1.3
O70404	Vesicle-associated membrane protein 8	Vamp8	2	6.0734	0.00493	1.3
Q61739	Integrin alpha-6	Itga6	3	11.8001	0.00020	1.3
P01027	Complement C3	C3	3	9.6830	0.00063	1.3
A2ARV4	Low-density lipoprotein receptor-related protein 2	Lrp2	5	22.3161	0.00409	1.3
P12382	ATP-dependent 6-phosphofructokinase, liver type	Pfkl	3	12.7193	0.00370	1.29
P11881	Inositol 1,4,5-trisphosphate receptor type 1	Itpr1	5	17.8101	3.12E-05	1.29
P28271	Cytoplasmic aconitate hydratase	Aco1	2	4.3600	0.00364	1.28
Q8VCT4	Carboxylesterase 1D	Ces1d	2	6.6092	0.01551	1.28
Q91YI0	Argininosuccinate lyase	Asl	2	8.7531	0.00032	1.27
Q64471	Glutathione S-transferase theta-1	Gstt1	2	5.5587	0.00013	1.26
Q6P1B1	Xaa-Pro aminopeptidase 1	Xpnpep1	3	7.8825	0.03278	1.26
P10852	4F2 cell-surface antigen heavy chain	Slc3a2	4	10.4098	0.00265	1.26
Q9WVK4	EH domain-containing protein 1	Ehd1	2	5.8015	0.00026	1.25
Q71LX4	Talin-2	Tln2	2	6.9617	0.01162	1.25
Q8VC30	Triokinase/FMN cyclase	Tkfc	3	10.8554	0.00073	1.25
P06281	Renin-1	Ren1	4	10.7058	0.00451	1.25
P63158	High mobility group protein B1	Hmgb1	2	6.2009	3.14E-05	1.24
Q9D0F9	Phosphoglucomutase-1	Pgm1	2	5.4388	0.00027	1.24
O88909	Solute carrier family 22 member 8	Slc22a8	3	9.1520	5.17E-06	1.24
Q3U4I7	Pyridine nucleotide-disulfide oxidoreductase domain-containing protein 2	Pyroxd2	3	10.0392	0.00151	1.24
Q9NYQ2	Hydroxyacid oxidase	Hao2	3	11.4539	0.02747	1.24
O70475	UDP-glucose 6-dehydrogenase	Ugdh	5	19.0586	0.00356	1.24
Q8BUV3	Gephyrin	Gphn	2	6.3220	0.00830	1.22
Q99K51	Plastin-3	Pls3	2	6.6408	0.01267	1.21
Q9DBM2	Peroxisomal bifunctional enzyme	Ehhadh	2	8.7039	0.01977	1.21
Q9Z1N5	Spliceosome RNA helicase Ddx39b	Ddx39b	2	6.8126	0.02905	1.21
Q8R0N6	Hydroxyacid-oxoacid transhydrogenase, mitochondrial	Adhfe1	3	10.8822	5.79E-06	1.21
P16546	Spectrin alpha chain, non-erythrocytic 1	Sptan1	3	11.5102	0.00302	1.21
Q99JY0	Trifunctional enzyme subunit beta, mitochondrial	Hadhb	4	13.9444	0.00113	1.2

**Table 2:** Proteomic identification of kidney proteins with a decreased abundance below 1.5-fold in the *mdx-4cv* model of Duchenne muscular dystrophy.

Accession	Protein	Gene	Unique peptides	Confidence score	Anova (p)	Max fold change
P48024	Eukaryotic translation initiation factor 1	Eif1	2	8.4814	0.00013	1.49
Q9JHR7	Insulin-degrading enzyme	Ide	2	5.2327	0.04111	1.46
P11862	Growth arrest-specific protein 2	Gas2	3	9.2866	4.11E-05	1.46
Q9DBG6	Dolichyl-diphospho-oligosaccharide-protein glycosyltransferase subunit 2	Rpn2	2	8.4270	0.00186	1.45
Q61838	Pregnancy zone protein	Pzp	9	29.2658	0.00065	1.42
Q61847	Meprin A subunit beta	Mep1b	2	7.1821	0.00095	1.4
P12658	Calbindin	Calb1	2	5.1746	0.01903	1.4
Q9CPY7	Cytosol aminopeptidase	Lap3	3	10.4237	0.02227	1.4
Q9DCY0	Glycine N-acyltransferase-like protein Keg1	Keg1	2	4.7402	0.00128	1.39
Q9CR67	Transmembrane protein 33	Tmem33	2	6.1302	0.00735	1.38
Q61207	Prosaposin	Psap	2	5.1656	0.00022	1.37
Q8JZZ0	UDP-glucuronosyltransferase 3A2	Ugt3a2	2	6.4221	0.02672	1.37
Q9DBX3	Sushi domain-containing protein 2	Susd2	4	14.5368	0.00470	1.37
Q9Z1J3	Cysteine desulfurase, mitochondrial	Nfs1	2	11.6958	1.28E-05	1.36
Q3U9G9	Lamin-B receptor	Lbr	2	7.4723	0.00013	1.36
Q9CQH7	Transcription factor BTF3 homolog 4	Btf3l4	2	8.2813	3.81E-05	1.34
Q9CYH2	Redox-regulatory protein FAM213A	Fam213a	2	6.9334	0.00034	1.34
Q05920	Pyruvate carboxylase, mitochondrial	*Pc*	2	5.6710	0.00120	1.32
Q05793	Basement membrane-specific heparan sulfate proteoglycan core protein	Hspg2	2	5.5448	0.00348	1.32
P12970	60S ribosomal protein L7a	Rpl7a	2	6.4383	0.01017	1.32
P51885	Lumican	Lum	2	7.6116	0.02121	1.32
Q00915	Retinol-binding protein 1	Rbp1	2	6.9546	0.00513	1.31
Q8BFW7	Lipoma-preferred partner homolog	Lpp	2	5.7683	0.01836	1.31
Q8QZT1	Acetyl-CoA acetyltransferase, mitochondrial	Acat1	2	8.9227	0.00886	1.3
O08638	Myosin-11	Myh11	13	44.8107	5.02E-05	1.3
P21107	Tropomyosin alpha-3 chain	Tpm3	2	7.0891	2.93E-05	1.29
Q99JB2	Stomatin-like protein 2, mitochondrial	Stoml2	2	7.7193	4.81E-05	1.29
Q32MW3	Acyl-coenzyme A thioesterase 10, mitochondrial	Acot10	3	11.9828	0.00043	1.28
P58774	Tropomyosin beta chain	Tpm2	2	7.4480	0.00049	1.27
P37804	Transgelin	Tagln	2	5.2768	0.01312	1.27
P21271	Unconventional myosin-Vb	Myo5b	2	6.2358	0.02245	1.27
P09103	Protein disulfide-isomerase	P4hb	2	5.3654	0.03115	1.27
Q9CR98	Protein FAM136A	Fam136a	2	5.8057	0.00522	1.26
P28653	Biglycan	Bgn	2	7.9254	0.00548	1.26
Q8BHN3	Neutral alpha-glucosidase AB	Ganab	3	8.1991	0.00178	1.26
Q02257	Junction plakoglobin	Jup	2	7.6926	0.01217	1.25
Q9D7X8	Gamma-glutamylcyclo-transferase	Ggct	2	7.4681	0.01870	1.25
Q8BJ64	Choline dehydrogenase, mitochondrial	Chdh	4	9.5358	0.00213	1.25
Q8CC88	von Willebrand factor A domain-containing protein 8	Vwa8	8	30.5625	0.01240	1.24
Q80X90	Filamin-B	Flnb	18	64.3416	0.00358	1.24
P51174	Long-chain specific acyl-CoA dehydrogenase, mitochondrial	Acadl	2	5.1602	0.00186	1.23
Q78IK4	MICOS complex subunit Mic27	Apool	2	5.9301	3.43E-06	1.22
Q06770	Corticosteroid-binding globulin	Serpina6	2	7.0902	0.00323	1.22
Q7TPR4	Alpha-actinin-1	Actn1	2	5.6749	0.00332	1.22
Q91X72	Hemopexin	Hpx	2	4.4037	0.03030	1.22
Q8QZY2	Glycerate kinase	Glyctk	3	12.7538	0.00018	1.22
Q61543	Golgi apparatus protein 1	Glg1	3	12.1393	0.00718	1.21
Q3V3R4	Integrin alpha-1	Itga1	4	11.2984	0.00068	1.21
Q8VDD5	Myosin-9	Myh9	15	57.2338	0.00070	1.21
Q8BL66	Early endosome antigen 1	Eea1	3	13.5801	5.59E-05	1.2
Q64727	Vinculin	Vcl	6	20.2663	0.00409	1.2

**Table 3:** List of mass spectrometrically identified high-molecular-mass mouse kidney proteins that are covered by a large number of unique peptide sequences.

Accession	Protein name	Gene	Peptides	Unique peptides	Coverage (%)	Molecular mass (kDa)
A2ARV4	Low-density lipoprotein receptor-related protein 2	Lrp2	187	187	56	518.9
P16546	Spectrin alpha chain, non-erythrocytic 1	Sptan1	158	158	72	284.4
Q62261	Spectrin beta chain, non-erythrocytic 1	Sptbn1	143	137	70	274.1
Q8VDD5	Myosin-9	Myh9	125	103	64	226.2
Q9JHU4	Cytoplasmic dynein 1 heavy chain 1	Dync1h1	184	184	50	531.7
Q9QXS1	Plectin	Plec	119	119	30	533.9
Q80X90	Filamin-B	Flnb	116	108	65	277.7
Q8BTM8	Filamin-A	Flna	108	99	60	281
P26039	Talin-1	Tln1	107	94	65	269.7
Q05793	Basement membrane-specific heparan sulfate proteoglycan core protein	Hspg2	103	103	46	398
Q9QXZ0	Microtubule-actin cross-linking factor 1	Macf1	99	97	19	831.4
Q68FD5	Clathrin heavy chain 1	Cltc	90	90	74	191.4
Q9JLB4	Cubilin	Cubilin	83	83	34	398.8
Q61001	Laminin subunit alpha-5	Lama5	84	84	38	403.8
O08638	Myosin-11	Myh11	89	68	50	226.9
Q61879	Myosin-10	Myh10	90	68	50	228.9
Q8CC88	von Willebrand factor A domain-containing protein 8	Vwa8	88	88	61	213.3
Q71LX4	Talin-2	Tln2	81	68	51	253.5

**Table 4:** List of mass spectrometrically identified mouse kidney marker proteins with a characteristic cellular localization.

Accession	Protein name	Gene	Coverage (%)	Unique peptides	Cellular localization
Q91X05	Podocin	Nphs2	18	4	Glomeruli podocytes
Q9QZS7	Nephrin	Nphs1	7	5	Glomeruli
P61148	Fibroblast growth factor FGF1	Fgf1	33	5	Glomeruli
Q9QXN5	Inositol oxygenase	Miox	74	12	Proximal tubule cytoplasm
O70577	Solute carrier family 22 member 2	Slc22a2	10	4	Proximal tubule basolateral membrane
O88909	Solute carrier family 22 member 8	Slc22a8	20	10	Proximal tubule basolateral membrane
Q60825	Sodium-dependent phosphate transport protein 2A	Slc34a1	28	12	Proximal tubule luminal membrane
Q8CFZ5	Solute carrier family 22 member 12	Slc22a12	19	12	Proximal tubule luminal membrane
O88576	Sodium-dependent neutral amino acid transporter B(0)AT3	Slc6a18	14	7	Proximal tubule luminal membrane
Q6A4L0	Solute carrier family 22 member 13	Slc22a13	12	6	Proximal tubule luminal membrane
Q91X17	Uromodulin	Umod	33	18	Distal tubule cytoplasm
O88335	ATP-sensitive inward rectifier potassium channel 1	Kcnj1	10	3	Distal tubule luminal membrane
P55014	Solute carrier family 12 member 1	Slc12a1	40	32	Distal tubule luminal membrane
P59158	Solute carrier family 12 member 3	Slc12a3	29	20	Distal tubule luminal membrane
P56402	Aquaporin-2	Aqp2	13	3	Collecting duct luminal membrane

**Table 5:** List of mass spectrometrically identified mouse kidney marker proteins with characteristic physiological functions in ion homeostasis.

Accession	Protein name	Gene	Coverage (%)	Peptides	Unique Peptides	Molecular mass [kDa]
P70414	Sodium/calcium exchanger 1	Slc8a1	6	5	5	108
Q8K596	Sodium/calcium exchanger 2	Slc8a2	4	2	1	100.6
Q8C3K6	Sodium/glucose cotransporter 1	Slc5a1	22	13	13	73.4
Q923I7	Sodium/glucose cotransporter 2	Slc5a2	16	10	9	73
Q8VDT1	Sodium/glucose cotransporter 4	Slc5a9	18	8	7	75
Q5SWY8	Sodium/glucose cotransporter 5	Slc5a10	20	8	8	64.7
G5E829	Plasma membrane calcium-transporting ATPase 1	Atp2b1	30	27	17	134.7
Q6Q477	Plasma membrane calcium-transporting ATPase 4	Atp2b4	25	22	12	133
Q8VDN2	Sodium/potassium-transporting ATPase subunit alpha-1	Atp1a1	50	47	31	112.9
Q6PIE5	Sodium/potassium-transporting ATPase subunit alpha-2	Atp1a2	21	17	1	112.1
Q6PIC6	Sodium/potassium-transporting ATPase subunit alpha-3	Atp1a3	24	17	2	111.6
P14094	Sodium/potassium-transporting ATPase subunit beta-1	Atp1b1	52	15	15	35.2
P97370	Sodium/potassium-transporting ATPase subunit beta-3	Atp1b3	19	4	4	31.8
O55143	Sarcoplasmic/endoplasmic reticulum calcium ATPase 2	Atp2a2	46	43	36	104.4
Q64518	Sarcoplasmic/endoplasmic reticulum calcium ATPase 3	Atp2a3	16	14	7	103.8
Q80XR2	Calcium-transporting ATPase type 2C member 1	Atp2c1	10	6	6	91.8
Q06138	Calcium-binding protein 39	Cab39	40	14	10	34.1
Q64374	Regucalcin	Rgn	33	8	8	29.9
P0DP27	Calmodulin-2	Calm2	72	10	10	14.9
Q9DB16	Calcium-binding protein 39-like	Cab39l	28	10	6	33.7
P12658	Calbindin	Calb1	67	20	20	30
P97816	Protein S100-G	S100g	71	8	8	9
P14211	Calreticulin	Calr	68	19	19	48
O08532	Voltage-dependent calcium channel subunit alpha-2/delta-1	Cacna2d1	17	14	14	110.3
Q02789	Voltage-dependent L-type calcium channel subunit alpha-1S	Cacna1s	1	1	1	188.0
Q921L3	Calcium load-activated calcium channel	Tmco1	12	2	2	21.2
Q60932	Voltage-dependent anion-selective channel protein 1	Vdac1	73	18	17	32.3
Q60930	Voltage-dependent anion-selective channel protein 2	Vdac2	57	11	11	31.7
Q60931	Voltage-dependent anion-selective channel protein 3	Vdac3	52	10	9	30.7
Q9EPK8	Transient receptor potential cation channel subfamily V member 4	Trpv4	15	9	9	98
O88335	ATP-sensitive inward rectifier potassium channel 1	Kcnj1	10	3	3	42.7
O88932	ATP-sensitive inward rectifier potassium channel 15	Kcnj15	6	3	3	42.6
Q9Z307	Inward rectifier potassium channel 16	Kcnj16	9	4	4	48
Q9WUB7	Chloride channel protein ClC-Ka	Clcnka	11	6	4	68.7
Q9WUB6	Chloride channel protein ClC-Kb	Clcnkb	13	6	4	68.7
Q99LI2	Chloride channel CLIC-like protein 1	Clcc1	12	4	4	53.9
Q9Z1Q5	Chloride intracellular channel protein 1	Clic1	74	14	14	27
Q9QYB1	Chloride intracellular channel protein 4	Clic4	71	15	15	28.7
Q8BXK9	Chloride intracellular channel protein 5	Clic5	57	11	9	28.3

**Table 6:** List of mass spectrometrically identified fatty acid binding proteins from mouse kidney.

Accession	Protein	Gene	Coverage (%)	Peptides	Unique Peptides	Molecular mass (kDa)
P12710	Fatty acid-binding protein FABP1, liver	Fabp1	43	5	5	14.2
P11404	Fatty acid-binding protein FABP3, heart	Fabp3	53	7	7	14.8
P04117	Fatty acid-binding protein FABP4, adipocyte	Fabp4	65	8	8	14.6
Q05816	Fatty acid-binding protein FABP5, epidermal tisssue	Fabp5	73	9	9	15.1
P51880	Fatty acid-binding protein FABP7, brain	Fabp7	14	1	1	14.9

**Table 7:** List of mass spectrometrically identified mouse kidney dystrophin and associated proteins.

Accession	Protein	Gene	Coverage (%)	Peptides	Unique Peptides	Molecular mass (kDa)
P11531	Dystrophin Dp140	Dmd	3	8	8	140
Q62165	Dystroglycan	Dag1	14	12	12	96.8
Q61234	Alpha-1-syntrophin	Snta1	22	8	8	53.6
Q61235	Beta-2-syntrophin	Sntb2	34	13	13	56.3
O70258	Epsilon-sarcoglycan	Sgce	3	1	1	49.7
Q9D2N4	Alpha-dystrobrevin	Dtna	3	2	1	84
O70585	Beta-dystrobrevin	Dtnb	16	6	5	74.4

## 2 Experimental design, materials, and methods

### 2.1 Biological samples and materials

The systematic cataloguing of the assessable kidney proteome was carried out with 12-month old wild type C57/BL6 mice and age-matched dystrophic *mdx-4cv* mice [9]. Animals were obtained from the Bioresource Unit of the University of Bonn [10] and kept under standard conditions and all procedures adhered to German legislation on the use of animals in experimental research.

For kidney protein extraction, protein digestion and the mass spectrometric analysis of the renal proteome, general materials and analytical grade reagents were purchased from Bio-Rad Laboratories (Hemel-Hempstead, Hertfordshire, UK), GE Healthcare (Little Chalfont, Buckinghamshire, UK) and Sigma Chemical Company (Dorset, UK). For the filter-aided sample preparation procedure, filter units Vivacon 500 (product number: VN0H22) were purchased from Sartorius (Göttingen, Germany). Protein digestion was carried out with sequencing grade modified trypsin from Promega (Madison, WI, USA). Pierce C18 spin columns and Acclaim PepMap 100 columns were from Thermo Fisher Scientific (Dublin, Ireland).

### 2.2 Kidney protein extraction and mass spectrometric analysis

Crude protein extracts were prepared as described in detail in the accompanying research paper [1]. The filter-aided sample preparation method was used as described by Wiśniewski et al. [11]. For the mass spectrometric establishment of the assessable mouse kidney proteome, peptide characterization was carried out by reverse-phased capillary high pressure liquid chromatography using an UltiMate 3000 nano system from Thermo Scientific [12]. The liquid chromatographic separation of peptides was coupled directly in-line with a Orbitrap Fusion Tribrid Mass Spectrometer from Thermo Scientific. Data analysis was carried out as previously described in detail [13].
